# MicroRNA-218 inhibits EMT, migration and invasion by targeting SFMBT1 and DCUN1D1 in cervical cancer

**DOI:** 10.18632/oncotarget.9850

**Published:** 2016-06-06

**Authors:** Zhaojing Jiang, Qiancheng Song, Rong Zeng, Jing Li, Jingyu Li, Xiaochun Lin, Xing Chen, Jiren Zhang, Yanfang Zheng

**Affiliations:** ^1^ Oncology Center, Zhujiang Hospital, Southern Medical University, Guangzhou, China; ^2^ Department of Cell Biology, School of Basic Medical Sciences, Southern Medical University, Guangzhou, China; ^3^ Department of Oncology, Xuzhou Cancer Hospital, Xuzhou, China; ^4^ Department of Pathology, Zhujiang Hospital, Southern Medical University, Guangzhou, China

**Keywords:** miR-218, EMT, cervical cancer, SFMBT1, DCUN1D1

## Abstract

Repeated infection with high-risk HPV is a major cause for the development and metastasis of human cervical cancer, even though the mechanism of the metastasis is still not completely understood. Here, we reported that miR-218 (microRNA-218) was downregulated in cervical cancer tissues, especially in metastatic cancer tissues. We found that miR-218 expression was associated with clinicopathological characteristics of patients with cervical cancer. MiR-218 overexpression inhibited Epithelial-Mesenchymal Transition (EMT), migration and invasiveness of cervical cancer cells *in vitro*. Moreover, miR-218 repressed the expression of SFMFBT1 (Scm-like with four MBT domains 1) and DCUN1D1 (defective in cullin neddylation 1, domain containing 1) by direct binding to the 3′UTRs of the mRNAs. The overexpression of SFMBT1 induced EMT and increased the migration and invasiveness of cervical cancer cells, while the overexpression of DCUN1D1 increased the migration and invasiveness of these cells, but did not induce EMT. An inverse correlation was observed between the expression of miR-218 and DCUN1D1 protein in cervical cancer tissues. Importantly, HPV16 E6 downregulated the expression of miR-218 in cervical cancer, while miR-218 rescued the promotion effect of HPV16 E6 on the expression of SFMBT1 and DCUN1D1. Taken together, our results revealed that HPV16 E6 promoted EMT and invasion in cervical cancer via the repression of miR-218, while miR-218 inhibited EMT and invasion in cervical cancer by targeting SFMBT1 and DCUN1D1.

## INTRODUCTION

Cervical cancer is the third most commonly diagnosed cancer and the fourth leading cause of cancer death in females worldwide [1]. The prognosis of patients with cervical cancer remains unsatisfactory, especially for those with advanced-stage tumors. Tumor metastases are responsible for approximately 90% of all cancer-related deaths [2]. Therefore, the identification of causes and potential markers of metastasis are important in order to promote an early diagnosis, predict the prognosis and develop novel therapeutic strategies for cervical cancer.

First discovered in 1993, microRNAs (miRNAs) are noncoding, single-stranded RNAs, 18-24 nucleotides in length, that are widely present in eukaryotes [3]. Recent studies have indicated that miRNAs are aberrantly expressed in different types of tumor tissues at various developmental stages and are considered to be master regulators of many important biological processes, including cell proliferation, apoptosis, and the stress response [4, 5]. Studies also have documented the role of miRNAs in metastasis, cellular migration, invasion, and EMT [6–8]. Compelling evidence shows that a number of miRNAs including miR-424, miR-29a, miR-375, participate in the invasion and metastasis of cervical cancer [9, 10].

Certain types of high-risk human papillomaviruses (HR-HPVs), such as HPV16 and HPV18, which are the most common cancer-related HPVs, have been recognized as major causative agents and are essential factors in the genesis and progression of cervical cancer [11, 12]. As reported, infection with HR-HPVs can cause aberrant expression of oncogenic and tumor-suppressive miRNAs [10, 13, 14]. Various miRNA genes are downstream targets of transcription factors such as c-Myc, p53, and E2F, and their expression can be directly or indirectly modulated by HPV oncogenic proteins E6 and E7, including miR-29a and miR-34a, which contribute to the initiation and progression of cervical cancer [10, 13, 14]. As reported, HPV-E6 binds p53 inducing its degradation by the ubiquitin–proteasome pathway inhibiting p53-dependent apoptosis activation, and HPV-E7 modifies pRb phosphorylation promoting cell cycle checkpoints escape and cell proliferation [15]. However, the mechanism by which HPV increases the development and metastasis of cancer is still uncertain.

Recent studies revealed that miR-218, as a tumor suppressor, was strongly downexpressed and related to proliferation, apoptosis and invasion in cervical cancer and some other cancers [16–19]. Some studies also demonstrated that the expression of miR-218 was strongly downregulated by HPV E6 [14, 20], but the mechanisms of the function of HPV on miR-218 and the role of miR-218 on the metastasis of cervical cancer are not well understood. In the present study, we attempted to further explore the role of miR-218 on the metastasis of cervical cancer. We found that miR-218 is downregulated in cervical cancer tissue and inhibits EMT, migration and invasiveness of cervical cancer cells, while HPV16 E6 induces EMT, and promotes invasiveness by repressing miR-218. Moreover, we identified SFMBT1 and DCUN1D1, which are responsible for the metastasis of cervical cancer, as the direct functional targets of miR-218.

## RESULTS

### Downregulation of miR-218 is correlated with the progression of cervical cancer and is associated with clinicopathologic characteristics

**Figure 1:**
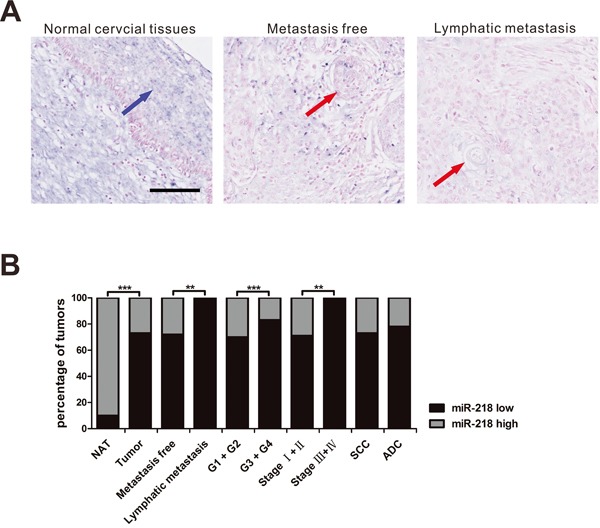
MiR-218 expression is lower in cervical cancer tissues *In situ* hybridization was used to detect miR-218 expression in a cervical cancer tissue microarray. **A.** Three representative cases of miR-218 expression are shown (scale bar, 100μm). Blue arrow demarcate normal cervical tissue; red arrows demarcate cervical cancer tissue. **B.** The percentage of specimens showing low or high miR-218 expression in relation to clinicopathologic parameters by Fisher's Exact Test. NAT, Cancer adjacent normal cervix tissue. (*P<0.05, **P<0.01, ***P<0.001.)

In order to identify the role of miR-218 in cervical cancer, we detected the expression of miR-218 in a cervical cancer tissue microarray that contained 94 cervical cancer tissues and 10 adjacent normal cervical tissues (Table 1). We found that the expression of miR-218 was significantly lower in cervical cancer tissues compared with adjacent normal cervical tissues (*P*<0.001) (Figure 1A). Then, we analyzed the association of miR-218 expression with clinicopathologic characteristics, including age, histology, clinical stage, differentiation and lymph node metastasis in 94 cervical cancer specimens. The results showed that lower miR-218 expression was significantly associated with advanced clinical stage (*P*= 0.006), poor tumor differentiation (*P*<0.001) and nodal metastasis (*P*= 0.003) (Figure 1B, Table 2). However, miR-218 expression was not significantly different between the various age groups and between patients with different tumor histologies. In total, the results demonstrate that miR-218 is downregulated in cervical cancer tissues, especially in advanced cervical cancer, and may be involved in the progression and metastasis of cervical cancer.

**Table 1:** Clinicopathologic characteristics of cervical cancer patients

Clinicopathologic variables	No. of cases (%)
Age	
<48	44 (46.8%)
≥48	50 (53.2%)
Histology	
Squamous cell carcinoma	85 (90.4)
Adenocarcinoma	9 (9.6%)
Differentiation	
well-differentiated	19 (20.2%)
moderately-differentiated	52 (55.3%)
poorly-differentiated	16 (17.0%)
undifferentiated	7 (7.5%)
Clinical stage	
I	73 (77.7%)
II	12 (12.8%)
III	7 (7.4%)
IV	2 (2.1%)
T classification	
T1	76 (80.9%)
T2	14 (14.9%)
T3	2 (2.1%)
T4	2 (2.1%)
N classification	
N0	89 (94.7%)
N1	5 (5.3%)

Clinicopathologic variables, including Histology, Differentiation, Clinical stage, T classification, N classification, are all followed the criteria of NCCN 2015 (National Comprehensive Cancer Network 2015).

**Table 2:** Correlation between the clinicopathologic features and expression of miR-218 in cervical cancer

Clinicopathologic characteristics		miR-218 expression		Total	*P*-value*
		Low	High		
Age^☒^	<48	33	11	44	0.865^a^
	≥48	36	14	50	
Histology	SCC	62	23	85	0.529
	ADC	7	2	9	
Clinical stage	Stage I+II	60	25	85	**0.006**
	Stage III+IV	9	0	9	
Differentiation	G1 + G2	50	21	71	**<0.001**
	G3 + G4	19	4	23	
Metastasis or not	Metastasis free	64	25	89	**0.003**
	Lymphatic metastasis	5	0	5	
Tumor	NAT	1	9	10	**<0.001**
	Cervical cancer tissue	69	25	94	

☒ the median age; SCC, Squamous cell carcinoma; ADC, Adenocarcinoma; G1, well differentiated; G2, moderately differentiated; G3, poorly differentiated; G4, undifferentiated; NAT, Cancer adjacent normal cervix tissue

a *χ^2^* Test

* Fisher's Exact Test.

### miR-218 inhibits EMT, migration and invasive ability of cervical cancer cells

**Figure 2:**
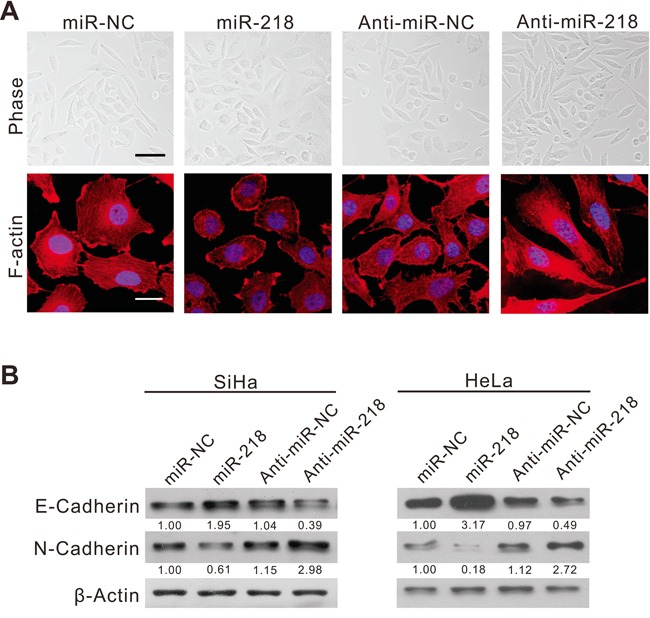
MiR-218 inhibits cervical cancer cell EMT *in vitro* **A.** Phase contrast microscopy (up, scale bar, 50μm), F-actin staining (down, scale bar, 20μm) of SiHa cells transfected with the negative control (NC), miR-218 mimics, anti-NC, or miR-218 inhibitor for 48 hours. DAPI (4′,6-diamidino-2-phenylindole) staining was used to detect nuclei and is merged with F-actin. **B.** Immunoblotting of E-cadherin and N-cadherin in SiHa and HeLa cells transfected with NC, miR-218 mimics, anti-NC, or miR-218 inhibitor. β-actin was used as a loading control.

To identify the role of miR-218 in cervical cancer metastasis, we transfected the cervical cancer cell lines SiHa and HeLa with synthesized miR-218 mimics or inhibitors. We found that miR-218 overexpression inhibited EMT in SiHa cells as shown by the changes in cell morphology and F-actin distribution (Figure 2A). At the protein level, cervical cancer cells transfected with miR-218 mimics led to a significant increase in the expression of E-cadherin, which is a typical protein expressed by epithelial cells (Figure 2B), and led to a decrease in the expression of N-cadherin, which is a typical protein expressed by mesenchymal cells (Figure 2B). In contrast, SiHa cells transfected with miR-218 inhibitor changed from a round shape to a spindle-like mesenchymal phenotype (Figure 2A). At the protein level, cervical cancer cells transfected with miR-218 inhibitor showed a decrease in the expression of E-cadherin and an increase in the expression of N-cadherin (Figure 2B). These results indicate that miR-218 can inhibit EMT in cervical cancer.

**Figure 3:**
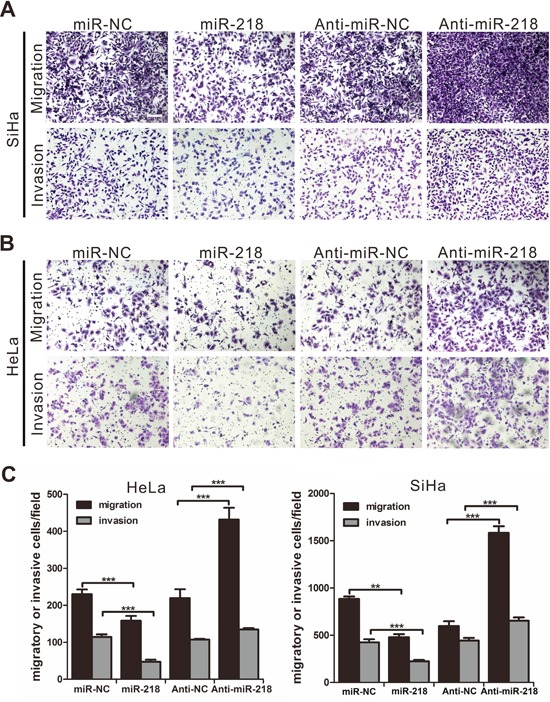
MiR-218 inhibits cervical cancer cell migration and invasiveness *in vitro* **A.** SiHa cells were transfected with NC, miR-218 mimics, anti-NC, or miR-218 inhibitor subjected to transwell migration and invasion assays (scale bar, 100μm). **B.** HeLa cells were transfected with NC, miR-218 mimics, anti-NC, or miR-218 inhibitor subjected to transwell migration and invasion assays. **C.** SiHa and HeLa cells transfected with miR-218 inhibitors showed a significant increase in cell migration and invasion. The results are representative of three independent experiments. (**P<0.01, ***P<0.001.).

The effect of miR-218 on cell motility and invasion was tested by Transwell assays. The results showed that miR-218 overexpression inhibited the migration and invasion of SiHa (Figure 3A and 3C) and HeLa (Figure 3B and 3C) cells as measured by crystal violet staining. Accordingly, cervical cancer cells transfected with miR-218 inhibitors showed a significant increase in cell migration and invasion.

These *in vitro* results suggest that miR-218 inhibits EMT, migration and invasiveness of cervical cancer cells.

### miR-218 directly targets the 3′UTRs of SFMBT1 and DCUN1D1

To determine the molecular basis of the effect of miR-218 on the progression and metastasis of cancer, we used PicTar [21] and TargetScan [22] software to identify putative target genes of miR-218. On the basis of the representation of miR-218 sites in their 3′untranslated regions (UTRs), over 100 mRNAs were predicted to be downregulated by miR-218. Among these candidates, eleven genes (ARID4B, BCAT1, CTNND2, DCUN1D1, DHX29, DOCK9, HAPLN1, LGR4, SFMBT1, SOCS7 and TAC1) were involved in the promotion of cancer progression and metastasis.

**Figure 4:**
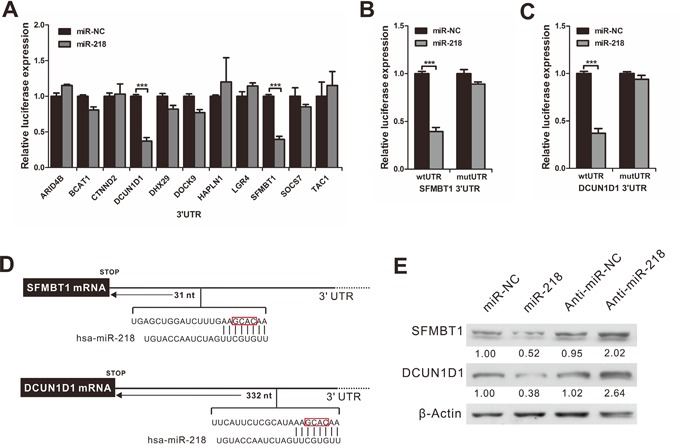
MiR-218 directly targets the SFMBT1 and DCUN1D1 3′UTR **A.** Luciferase activity in SiHa cells transfected with miR-218 mimics or NC after transfection of the indicated 3′UTR-driven reporter constructs. **B.** Mutation in the putative binding sites of miR-218 in SFMBT1 3′UTRs abrogated the responsiveness to miR-218. Wt, wild-type; mut, mutation at 49–52 nt of the SFMBT1 3′UTR; **C.** Mutation in the putative binding sites of miR-218 in DCUN1D1 3′UTRs abrogated the responsiveness to miR-218. Wt, wild-type; mut, mutation at 349–352 nt of the DCUN1D1 3′UTR. **D.** The putative miR-218-binding site in the SFMBT1 and DCUN1D1 3′UTR. **E.** The protein levels of SFMBT1 and DCUN1D1 were determined by Western blot analyses after transfection with NC, miR-218 mimics, anti-NC, or anti-miR-218 in SiHa cells. β-actin was used as a loading control.

To determine whether miR-218 targets these genes directly, we constructed vectors that contained the 3′UTR of each of the eleven putative miR-218 targets and a luciferase construct. Reporter assays revealed that miR-218 significantly repressed two of the UTRs: SFMBT1 and DCUN1D1 (Figure 4A) in miR-218-overexpressing SiHa cells. Furthermore, mutations in the putative binding sites of miR-218 in these two 3′UTRs abrogated their responsiveness to miR-218 (Figures 4B, 4C and 4D).

In accordance with these results, we found a clear decrease in endogenous SFMBT1 and DCUN1D1 protein expression in cervical cancer cells when miR-218 was overexpressed (Figure 4E). In addition, transfection with miR-218 inhibitor significantly increased the expression of SFMBT1 and DCUN1D1 protein in SiHa cells (Figure 4E). These results suggest that miR-218 can downregulate SFMBT1 and DCUN1D1 expression by directly targeting their 3′UTRs.

### A decrease of SFMBT1 and DCUN1D1 is required for miR-218-mediated inhibition of motility and invasiveness of cervical cancer cells

**Figure 5:**
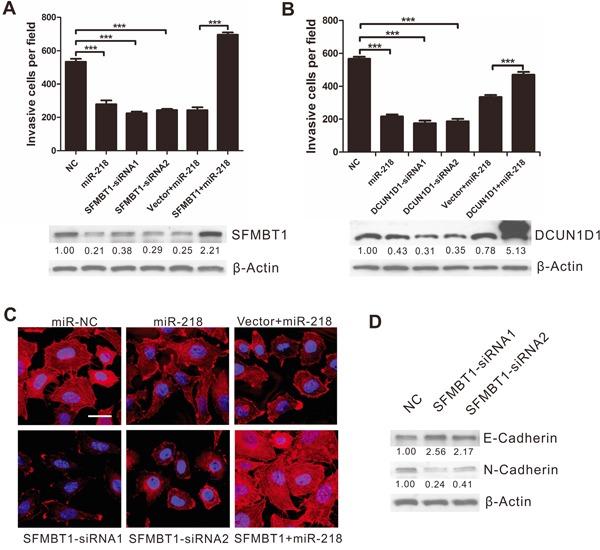
MiR-218 overexpression and SFMBT1 inhibition produce similar changes, which are restored by SFMBT1 ectopic expression *in vitro* **A.** Transwell invasion assays of SiHa cells were performed after transfection with NC, siRNA against SFMBT1, and/or miR-218 as indicated (up); SFMBT1 protein were detected by Western blot analysis (down). **B.** Transwell invasion assays of SiHa cells were performed after transfection with NC, siRNA against DCUN1D1, and/or miR-218 as indicated (up); DCUN1D1 protein were detected by Western blot analysis (down). The results are representative of at least three independent experiments. **C.** F-actin staining of SiHa cells transfected as indicated (scale bar, 20μm). **D.** SiHa cells were transfected with SFMBT1 siRNAs, and then E-cadherin and N-cadherin protein levels were detected by Western blot analysis. β-actin was used as a loading control.

To determine whether SFMBT1 and DCUN1D1 serve as critical mediators in the role of miR-218 on cervical cancer, we knocked down SFMBT1 and DCUN1D1 by the application of synthetic siRNA in SiHa and HeLa cells. Western blot analysis showed that the siRNA effectively reduced SFMBT1 and DCUN1D1 protein levels (Figure 5A and 5B). The results showed that knockdown of SFMBT1 or DCUN1D1 inhibited invasion of cervical cancer cell similarly as those that were observed in miR-218 overexpression treatment (Figure 5A and 5B). To determine whether these effects depend specifically on SFMBT1 or DCUN1D1 suppression, we used an expression construct that encodes the entire SFMBT1 or DCUN1D1 coding sequence and lacks the 3′UTR. The results showed that the ectopic expression of SFMBT1 nearly completely rescued the miR-218-mediated suppression of invasion in miR-218-overexpressing cells (*P*<0.001, Figures 5A), while re-expression of DCUN1D1 partially rescued the miR-218-mediated suppression of invasion (*P*<0.001, Figures 5B). This suggests that the decrease in SFMBT1 and DCUN1D1 expression is required for the decrease in the invasiveness of miR-218-overexpressing cervical cancer cells.

### miR-218 inhibits cancer cell EMT via the inhibition of SFMBT1

Next, we asked whether SFMBT1 or DCUN1D1 is involved in the relationship between miR-218 and EMT. Surprisingly, we found that SiHa cells showed less spindle-like mesenchymal phenotype when SFMBT1 was repressed (Figure 5C), which mimics the effect of miR-218 on EMT. SFMBT1 overexpression rescues the inhibitory effect of miR-218 on EMT with more pindle-like mesenchymal phenotype in miR-218-overexpressing SiHa cells. In contrast, DCUN1D1 did not lead to these same changes (Supplementary Figure S1). In addition, the abolition of SFMBT1 significantly increased E-cadherin expression and decreased the N-cadherin expression (Figure 5D), while the abolition of DCUN1D1 did not (Supplementary Figure S1). These results indicate that the ability of miR-218 to inhibit EMT is attributable to it's capacity of inhibiting SFMBT1.

### Clinical associations of miR-218 and DCUN1D1 expression

**Figure 6:**
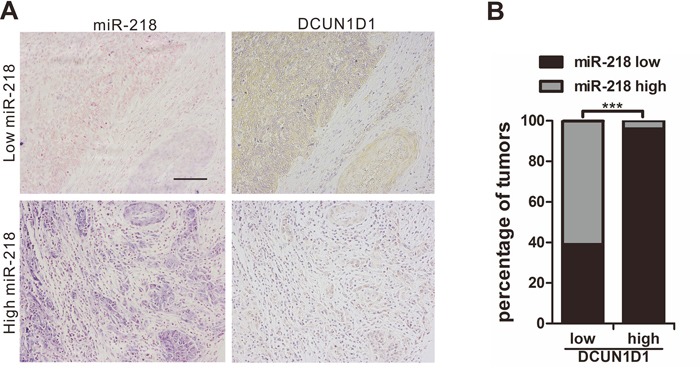
Clinical associations of miR-218 with DCUN1D1 expression **A.** Immunostaining shown that DCUN1D1 expression is negatively correlated with miR-218 expression (scale bar, 100μm). The upper 2 photos are from a stage IV case, while the lower 2 photos are from a stage I case. **B.** Most specimens with high DCUN1D1 expression have low miR-218 expression, while more specimens with low DCUN1D1 expression have high miR-218 expression (^***^, *P*<0.001).

To study the relationship between miR-218 and its target genes, we detected the expression of DCUN1D1 in another cervical cancer tissue microarray. The results showed that DCUN1D1 expression by immunostaining is negatively correlated with miR-218 expression in cervical cancer tissues (*P*<0.001, Figure 6A and 6B, Table 3). DCUN1D1 isotype control staining was performed on cervical cancer tissue in order to assure specificity (Supplementary figure S2). Then, we analyzed the association between DCUN1D1 expression and clinicopathologic features, and the results revealed that the expression of DCUN1D1 was significantly higher in cervical cancer tissues than in adjacent normal cervical tissues (*P*= 0.017). Furthermore, the results demonstrated a positive correlation between DCUN1D1 expression and clinical stage (*P*= 0.009) as well as a positive correlation between DCUN1D1 expression and differentiation (*P*= 0.010, Table 4). We failed to find a relationship between DCUN1D1 expression and tumor metastasis, which may be due to an insufficient number of cervical cancer samples. These results further implicate the important role of miR-218 in the development and metastasis of cervical cancer through DCUN1D1. Without an available antibody for immunohistochemistry, this study failed to detect SFMBT1 expression in cervical cancer tissues.

**Table 3:** Correlation between the expression of miR-218 and target genes

*Spearman*'s correlation	DCUN1D1	miR-218
**DCUN1D1**	1	−0.633
***P***		<0.001
N	94	

**Table 4:** Correlation between the clinicopathologic features and expression of DCUN1D1 in cervical cancer

Clinicopathologic characteristics		DCUN1D1 expression		Total	*P*-value*
		Low	High		
Age	<48	21	23	44	0.176^a^
	≥48	17	33	50	
Histology	SCC	33	52	85	0.331
	ADC	5	4	9	
Clinical stage	Stage I+II	37	48	85	**0.009**
	Stage III+IV	0	9	9	
Differentiation	G1 + G2	34	37	71	**0.010**
	G3 + G4	4	19	23	
Metastasis or not	Metastasis free	38	51	89	0.058
	Lymphatic metastasis	0	5	5	
Tumor	NAT	7	3	10	**0.017**
	Cervical cancer tissue	38	56	94	

SCC, Squamous cell carcinoma; ADC, Adenocarcinoma; G1, well differentiated; G2, moderately differentiated; G3, poorly differentiated; G4, undifferentiated; NAT, Cancer adjacent normal cervix tissue

a *χ^2^* Test

* Fisher's Exact Test.

### HPV16 E6 induces EMT and promotes invasiveness of cervical cancer via the repression of miR-218 expression

**Figure 7:**
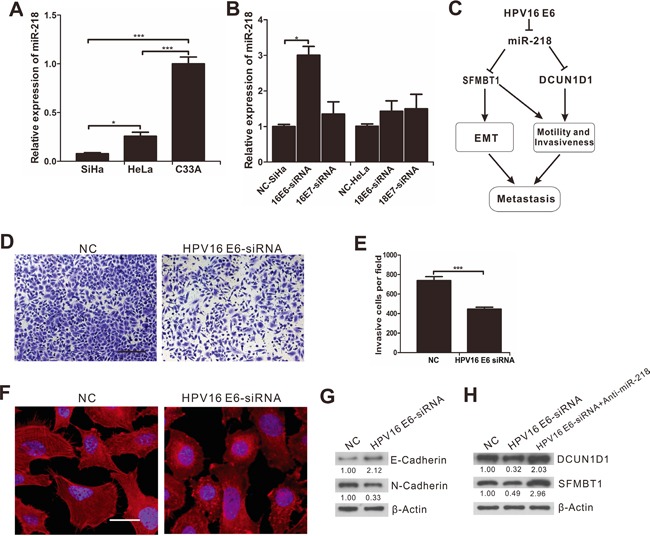
The inhibition of miR-218 on EMT, migration and invasion of cervical cancer cells downregulated by HPV16 E6 **A.** qRT-PCR analysis of miR-218 expression in SiHa (HPV16^+^), HeLa (HPV18^+^) and C33A (HPV^−^) cells. **B.** qRT-PCR analysis of miR-218 expression in SiHa or HeLa cells transfected with NC or siRNAs as indicated. RT-PCR data were normalized to β-actin. Experiments were performed three times, and the data are presented as the mean±SEM. **C.** A graphical representation illustrates the role of the miR-218-mediated pathway in cervical cancer metastasis. **D.** and **E.** Transwell invasion assays showed SiHa cells transfected with HPV16 E6 siRNA had decreased invasiveness compared with NC (scale bar, 100μm). **F.** F-actin staining of SiHa cells transfected with HPV16E6 siRNA or negative control (scale bar, 20μm). **G.** and **H.** SiHa cells were transfected with NC or siRNAs, then E-cadherin, N-cadherin, SFMBT1 and DCUN1D1 proteins were detected by Western blot analysis. β-actin was used as a loading control.

In order to identify the effect of HPV on miR-218 expression, we detected the expression of miR-218 in cervical cancer cells that are infected with different types of HPV. The results showed that the expression of miR-218 was significantly decreased in SiHa (HPV16^+^) (*P*<0.001) and HeLa (HPV18^+^) (*P*<0.001) cells compared with C33A (HPV^−^) cells (Figure 7A). HPVs contain two oncogenes (E6 and E7) that are involved in cellular transformation and play important roles in carcinogenesis [23]. Therefore, we knocked down HPV16 E6, HPV16 E7, HPV18 E6 and HPV18 E7 using synthetic siRNA in SiHa or HeLa cells to identify the most important oncogenic component in HPV that downregulate miR-218 expression (Figure 7B and Supplementary Figure S3). We found that the expression of miR-218 was significantly upregulated only in the cervical cancer cells transfected with synthetic siRNA against HPV16 E6 (*P*<0.05). The results showed that HPV16 E6 is the most important HPV oncoprotein that is involved in the repression of miR-218 in cervical cancer cells (Figure 7B).

Next, we explored the interaction between HPV16 E6 and miR-218 in EMT and invasiveness of cervical cancer. The results showed that the abolishment of HPV16 E6 reversed EMT and decreased the ability of cervical cancer cells to invade (Figures 7D, 7E, 7F and 7G). To determine whether the inhibition of EMT and invasion after the knockdown of HPV16 E6 in cervical cancer cells depends on miR-218 upregulation, we transfected both a synthetic siRNA against HPV16 E6 and a miR-218 inhibitor into SiHa cells. The results showed that inhibition of HPV16 E6 downregulated SFMBT1 and DCUN1D1 and that downregulation of miR-218 rescued the inhibitory effect of the knockdown of HPV16 E6 on the expression of SFMBT1 and DCUN1D1(Figure 7H). These results indicate that HPV16 E6 promotes EMT and invasion in cervical cancer via the repression of miR-218, while miR-218 inhibits EMT and invasion in cervical cancer by targeting SFMBT1 and DCUN1D1.

Collectively, our findings indicate that HPV16 E6 decreases miR-218 and upregulates its targets, which plays an important role in the metastasis of cervical cancer. We show a graphical representation illustrating the role of the miR-218-mediated pathway in cervical cancer metastasis (Figure 7C).

## DISCUSSION

More than 90% of carcinoma-associated mortality is caused by distant metastases rather than by the primary tumors [2]. Cancer metastasis is a complex process that requires many molecular and cellular events. Repeated infections with high-risk HPV strains are the prerequisite for the development of human cervical cancer [11, 12]. Infection with high-risk HPV also promotes the metastasis of some cancers [24, 25], but the mechanism is still not clearly understood.

miR-218 is encoded within intronic sequences of SLIT2 and SLIT3, which act as a tumor suppressor gene in many cancers [26]. Recently, miR-218 has been shown to be downregulated in cancers and this is associated with poor prognosis [16, 18]. As reported, miR-218 sensitizes cancer cells to apoptosis and inhibits tumorigenicity and migration in some cancers through different targets, such as IKK-beta and Robo1 receptor [27, 28]. Despite the tumor suppressive role of miR-218 has been implicated in previous studies, the role of miR-218 in tumor metastasis and its molecular mechanism is not fully understood. In this study, we show that miR-218 was decreased in cervical cancer. Moreover, the expression of miR-218 was lower in malignant tumors compared with benign tumors and were negatively correlated with the clinical stage and metastasis of cervical cancer. Our study shows that miR-218, as a tumor suppressor miRNA, also is a negative regulator of the pro-metastasizing genes SFMBT1 and DCUN1D1.

SFMBT1 belongs to the malignant brain tumor (MBT) domain-containing protein family, which contributes to multiple cellular processes, including cell proliferation [29] and maintenance of the characteristics of stem cells [30]. Recent studies have indicated that SFMBT1 functions as a candidate promoter of metastasis and is involved in a critical LSD1-SFMBT1-dependent chromatin regulatory program. This program is exploited by Snail, induces EMT and contributes to cancer progression and metastasis [31]. EMT is a vital developmental program and contributes to malignant progression and metastasis [32]. The role of SFMBT1 in cervical cancer has not been reported in previous studies. Our results showed that SFMBT1, as the direct and functional target of miR-218, promotes EMT and invasion in cervical cancer, which establishes SFMBT1 as a critical promoter of metastasis. Considering our own findings as well as those that have been previously published, we speculate that miR-218 might inhibit the invasion and metastasis of cervical cancer by targeting SFMBT1.

DCUN1D1 is an oncogene that increases the efficiency of neddylation, which may lead to a series of diseases, including cancers [33, 34]. Increasing evidence revealed that DCUN1D1 is overexpressed in many types of malignant tumors[35] and regulates tumor progression, chemosensitivity, angiogenesis and metastasis [36, 37]. Increased DCUN1D1 promotes regional lymph node and brain metastases, and decreases the survival of patients with lung squamous cell carcinoma [37]. In this study, we revealed that DCUN1D1 is upregulated in human cervical cancer and is positively correlated with cervical cancer grade and clinical stage. Interestingly, we found that DCUN1D1 was targeted by miR-218 and knockdown of DCUN1D1 significantly decreased the invasiveness of cervical cancer cells. Our results imply that DCUN1D1 may play a vital role in the inhibitory effects of miR-218 on cervical cancer metastasis.

Viral integration may greatly modulate the host cell genome and affect the expression of genes that are located nearby. A significant number of miRNAs are located in fragile sites that are close to HPV integration sites [38]. Thus, the miRNA expression profile in human cervical cancers may be deregulated by HPVs. Recent reports have suggested that oncogenic HPV E6 may interrupt the expression of various miRNAs, such as miR-34a [13] and miR-218 [14]. In this study, we identified that it is HPV16 E6 responsible for the decrease of miR-218 expression in cervical cancer cells. Moreover, we found that HPV16 E6 increases the expression of SFMBT1 and DCUN1D1 and miR-218 rescues the function of HPV16 E6. It was observed that the presence of high-risk HPVs is associated with an increased propensity for vascular space invasion, lymph node metastasis and enlarged tumor size in some cancers [24, 25, 39]. Here, we found that HPV16 E6 promotes EMT and enhances the invasiveness of cervical cancer cells, which is consistent with the effects of SFMBT1 and DCUN1D1 and is contrary to the effect of miR-218 on cervical cancer metastasis. In total, we suggest that HPV16 E6 may promote the metastasis of cervical cancer by repressing miR-218 expression, and then promote the expression of SFMBT1 and DCUN1D1. Therefore, this may be one of the mechanisms that are responsible for the cervical cancer metastasis. However, in HeLa cells with HPV18 infection, it appears that neither HPV18E6 nor HPV18E7 affects miR-218 expression. As we known, microRNAs can regulate many target genes *in vivo*, and also can be regulated by more than one upstream genes. Thus, we think that miR-218 may be repressed by some other genes instead of HPV18E6 or HPV18E7 in HeLa cells, which is an interesting issue to be explored in the future.

In the present study, we demonstrated the role of miR-218 and its mechanism of inhibiting cervical cancer metastasis. Our findings identify a new mechanism of HPV16 E6 promoting EMT and metastasis by repressing miR-218, and then upregulating SFMBT1 and DCUN1D1. Because distant metastasis is responsible for patient mortality in the vast majority of cases of human carcinoma, miR-218 might become a key and potential prognostic factor and a candidate target for therapy in patients with cervical cancer.

## MATERIALS AND METHODS

### In situ hybridization and immunohistochemistry of human cervical cancer tissue microarrays

Cervical cancer tissue microarrays (CR2083; Shanghai Outdo Biotech, China) were constructed with 104 formalin-fixed, paraffin-embedded cervical cancer tissues and corresponding adjacent normal cervical tissues. The protocol for the detection of miRNAs by *in situ* hybridization (ISH) has been previously published [40]. The sequence of the probes (Exiqon, Vedbaek, Denmark) for the hsa-miR-218 containing the locked nucleic acid/digoxigenin–modified bases was as follows: ACATGGTTAGATCAAGCACAA. Immunohistochemical staining for proteins was performed as previously described [41, 42]. The intensity of the miR-218 and protein staining in epithelial cells of the 104 cervical cancer samples was scored according to a semiquantitative scale as previously described [41, 42]. Briefly, immunostaining was defined as “high” if the immunoreactivity was observed in 10% or more of the cells in paraffin sections; tumors with lower percentages of immunoreactive cells were deemed to have “low” expression.

### Cell culture

The human cervical cancer cell lines SiHa, HeLa and C33A were purchased from the American Type Culture Collection (Manassas, VA, USA). The cells were cultured in Dulbecco's Modified Eagle's Medium supplemented with 10% FBS (Invitrogen-GIBCO, Grand Island, NY, USA) and incubated at 37°C in 5% CO_2_. They had been passed for less than 6 months in culture when the experiments were performed. SiHa, HeLa and C33A cells are HPV16-positive, HPV18-positive, and HPV-negative, respectively. The cell lines were characterized by DNA analysis using short tandem repeat finger printing.

### Real-time quantitative analysis

Total RNA, inclusive of the small RNA fraction, was extracted from cultured cells with TRIzol reagent (TaKaRa Otsu, Shiga, Japan) according to the manufacturer's instructions. cDNA was synthesized with the M-MLV reverse transcriptase (Invitrogen, Grand Island, NY, USA) for miR-218 or with the PrimeScript™ RT reagent Kit with gDNA Eraser (TaKaRa Otsu, Shiga, Japan) for HPV E6/E7. Real-time PCR was performed in a Real-Time Thermocycler 7500 (Applied Biosystems, Foster City, CA, USA) with a SYBR Green I Real-Time PCR Kit (GenePharma, Shanghai, China) for miR-218 or with a SYBR Premix Ex TaqTM (Tli RNaseH Plus) (TaKaRa Otsu, Shiga, Japan) for HPV E6/E7. For normalization, U6 and β-actin were used as the endogenous controls for miR-218 and HPV E6/E7, respectively. The relative expression levels of miRNAs and mRNAs in each sample were tested in triplicate and were calculated and quantified with the 2^−ΔΔCt^ method after normalization for expression of the positive control. The oligonucleotide sequences of the miRNA mimics, the miRNA inhibitors, those used for gene knockdown experiments, the gene cloning primers, the 3′UTR cloning primers, and the 3′UTR mutagenesis primers are summarized in Supplementary Table S1.

### Invasion and motility assays

For the motility assays, 1.0×10^5^ cells were placed in the top chamber of each insert (BD Biosciences, San Jose, CA, USA) with 8.0-mm pores; for the invasion assays, 2.0×10^5^ cells were seeded in a chamber (BD Biosciences) pre-coated with 0.2% Matrigel (Collaborative Research, Boston MA, USA). The cells were seeded in serum-free media and 10% fetal bovine serum was added to the culture medium in the lower chamber as a chemoattractant. After 24 hours of incubation at 37°C, the cells that remained in the upper compartment were removed by cotton swabs, and those that had invaded through the membrane were stained with a dye solution containing 20% methanol and 0.1% crystal violet. The cells were then imaged under a light microscope (Olympus) and ten individual fields were counted per insert. The results are presented as an average of three separate experiments.

### Immunoblots

The cells were lysed in 2×SDS sample buffer that contained a protease inhibitor cocktail (Roche, Indianapolis, IN, USA) on ice; the cell debris was then removed by centrifugation. Equal amounts of protein were analyzed by SDS–PAGE, transferred to a polyvinylidene difluoride membrane, and then probed with antibodies against β-actin (Beijing Ray Antibody Biotech, Beijing, China), SFMBT1 and DCUN1D1 (Abcam, Cambridge, UK), E-cadherin, and N-cadherin (Cell Signaling Technology, Boston, MA, USA) at 4°C overnight. After incubation with the primary antibodies, the membranes were washed with TBS/0.05% Tween-20 and incubated with horseradish peroxidase–conjugated secondary antibodies at room temperature for 1 hour. Proteins were detected by enhanced chemiluminescence substrates (Gene). The protein levels were normalized to β-actin expression.

### Luciferase assays

HEK293 cells were transfected with the indicated psiCHECK-2 luciferase construct. All cells were also transfected with miRNA mimics or with the negative control. Lysates were collected 36 hours after transfection and luciferase activities were measured by a dual-luciferase reporter assay system (Promega, Madison, WI, USA).

### Vector constructs

The 3′UTR of Rictor, which encompasses the predicted miRNA sequences, was inserted into the multiple cloning site of the reporter vector psiCHECK-2 (Promega) with XhoI and NotI. The seed-region-mutated reporters contained engineered point mutations of four nucleotides complementary to the 5′end of the relevant miRNA. The primers used for cloning are listed in Supplementary Table 1. All sequences were confirmed by Sanger sequencing.

### Statistical analysis

Statistical analysis was performed with the SPSS statistical software program (Version 13.0; SPSS Inc.). The association between miR-218 and protein expression was analyzed by the *χ^2^* test or Fisher's Exact Test. Differences between groups were analyzed by the Student t test or one-way ANOVA, or if the data were not distributed normally, the non-parametric Mann–Whitney test was used. A correlation analysis was performed using the *Spearman* correlation analysis. *P*<0.05 was considered statistically significant. Data are presented as the mean ± SEM.

## SUPPLEMENTARY FIGURES AND TABLE




