# Supplementary material for: MicroRNA-218 inhibits EMT, migration and invasion by targeting SFMBT1 and DCUN1D1 in cervical cancer
Source: Oncotarget. 2016 Jun 6;7(29):45622–36. doi: 10.18632/oncotarget.9850 (PMC5216747; doi:10.18632/oncotarget.9850)
Supplement: Supplementary file 1 [file oncotarget-07-45622-s001.pdf]

SUPPLEMENTARY FIGURES AND TABLE

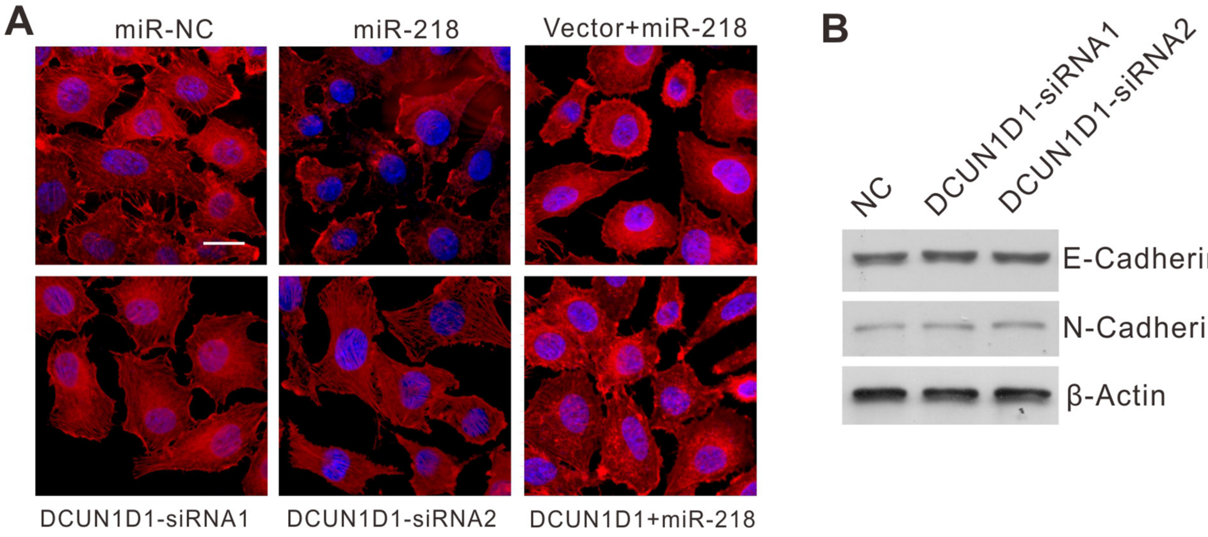

Supplementary Figure S1: The effect of DCUN1D1 on EMT of cervical cancer cells. A. F-actin staining microscopy of SiHa cells transfected as indicated (scale bar, 20μm). **B.** SiHa cells were transfected with DCUN1D1 siRNAs, then E-cadherin and N-cadherin protein levels were detected by Western blot analysis. β-actin was used as a loading control.

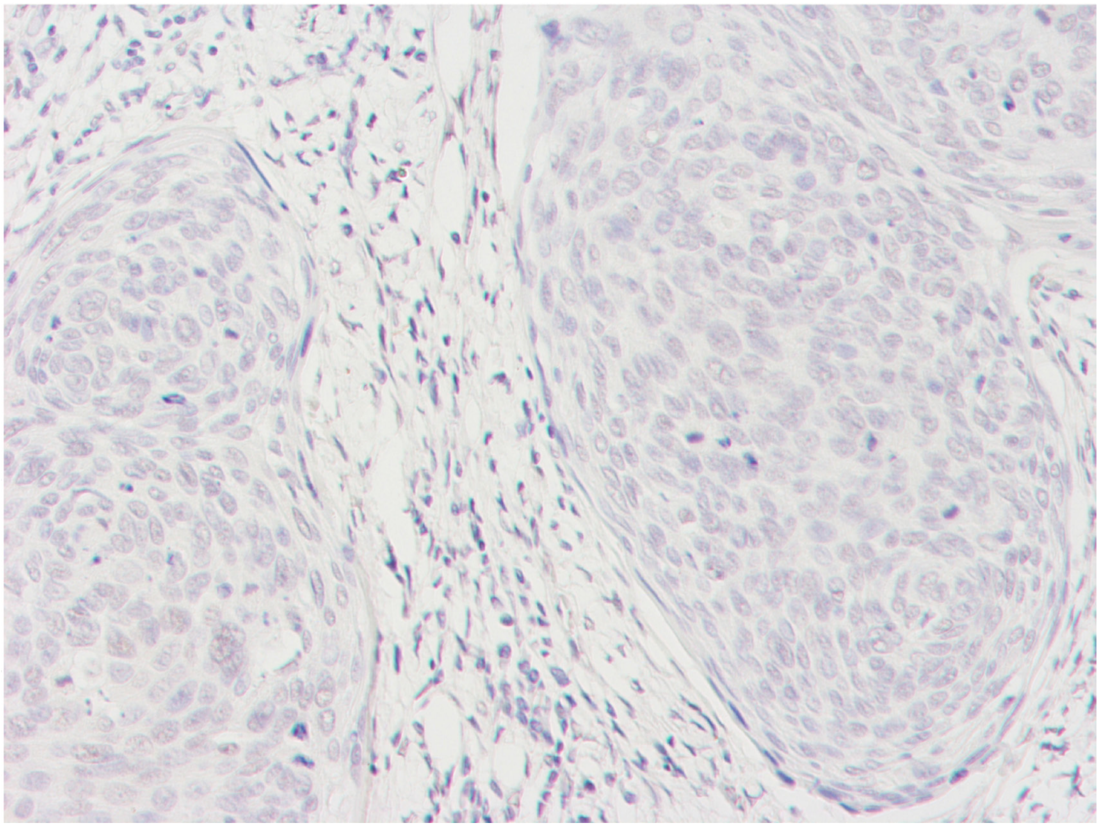

Supplementary Figure S2: DCUN1D1 isotype control staining in cervical cancer tissue.

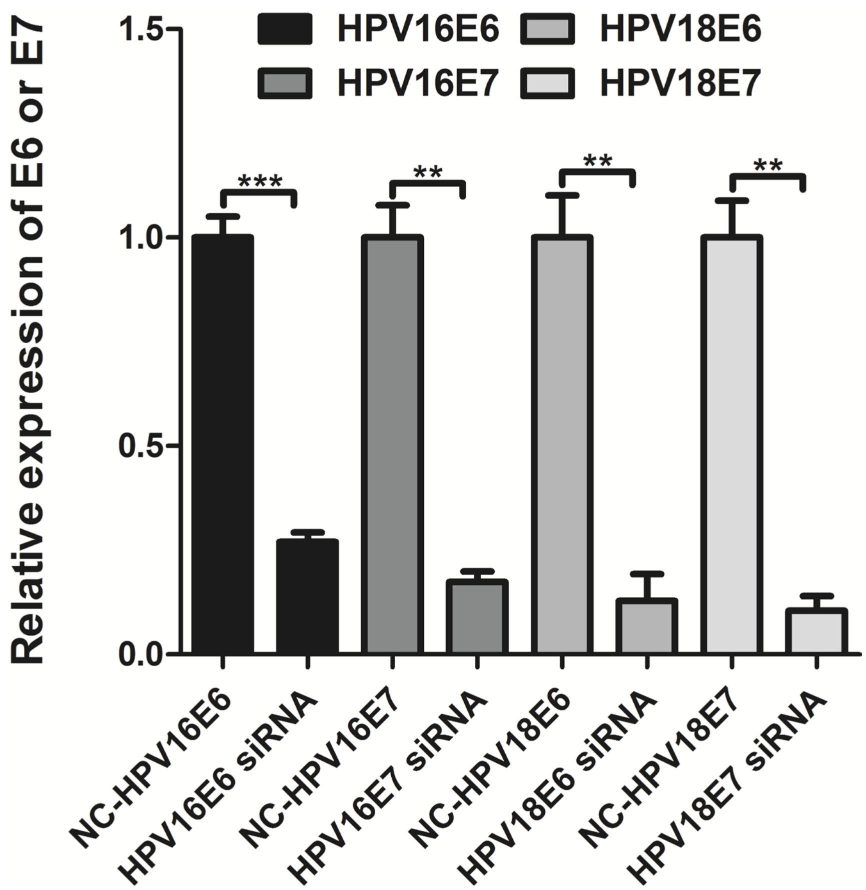

Supplementary Figure S3: The expression of HPV E6 or E7 in SiHa or HeLa cells transfected with specific siRNA. qRT-PCR analysis of HPV E6 or E7 expression in SiHa or HeLa cells transfected with negative control (NC) or siRNAs as indicated. qRT-PCR data were normalized to β-actin. Experiments were repeated in triplicate, and the data are presented as the mean ± SEM. Statistical analysis was performed with the Student *t* test.

Supplementary Table S1: Oligonucleotides used in this study

See Supplementary File 1
