# Supplementary material for: MicroRNA-218 inhibits EMT, migration and invasion by targeting SFMBT1 and DCUN1D1 in cervical cancer
Source: Oncotarget. 2016 Jun 6;7(29):45622–36. doi: 10.18632/oncotarget.9850 (PMC5216747; doi:10.18632/oncotarget.9850)
Supplement: Supplementary file 2 [file oncotarget-07-45622-s002.doc]

**Supplementary Table S1**

**Oligonucleotides used in this study**

| **Sequence Name** | **Sequence (5’ to 3’)** |
| --- | --- |
| **miRNA mimics and inhibitors** |  |
| RNA duplex control (sense) | UUCUCCGAACGUGUCACGUTT |
| miR-218 mimics (sense) | UUGUGCUUGAUCUAACCAUGU |
| miRNA inhibitor NC | CAGUACUUUUGUGUAGUACAA |
| miR-218 inhibitor | ACAUGGUUAGAUCAAGCACAA |
| **Gene knockdown** |  |
| siRNA-DCUN1D1-1 (sense) | UUGUGCUUGAUCUAACCAUGU |
| siRNA-DCUN1D1 -2 (sense) | GUUCAGAGCAGCAACACAGTT |
| siRNA-SFMBT1-1 (sense) | CUGGGAAGAUUAUCUAGAATT |
| siRNA-SFMBT1-2 (sense) | GGCUGAAGCUACGUUAUGATT |
| siRNA-HPV16 E6 (sense) | GAGGUAUAUGACUUUGCUUTT |
| siRNA-HPV16 E7 (sense) | AGGAGGAUGAAAUAGAUGGTT |
| siRNA-HPV18 E6 (sense) | CACUUCACUGCAAGACAUATT |
| siRNA-HPV18 E7 (sense) | CCACAACGUCACACAAUGUTT |
| **Gene cloning primer** |  |
| SFMBT1-F-KpnI | CGGGGTACC ATGAACGGGGAGCAGCAGCT |
| SFMBT1-R-NotI | ATTTGCGGCCGCTCAGTTGGCAAACTGCTCAT |
| DCUN1D1-F-HindIII | CCCAAGCTT ATGAACAAGTTGAAATCATC |
| DCUN1D1-R-NotI | ATTTGCGGCCGC CTACACTGTTGTACTTTTTGT |
| **QRT-PCR primer** |  |
| HPV18-E6-F | TGGCGCGCTTTGAGGA |
| HPV18-E6-R | TGTTCAGTTCCGTGCACAGATC |
| HPV18-E7-F | TGTATTGCATTTAGAGCCCCAAA |
| HPV18-E7-R | CTTCCTCTGAGTCGCTTAATTGC |
| HPV16-E6-F | CTGCAATGTTTCAGGACCCA |
| HPV16-E6-R | TCATGTATAGTTGTTTGCAGCTCTGT |
| HPV16-E7-F | AAGTGTGACTCTACGCTTCGGTT |
| HPV16-E7 -R | GCCCATTAACAGGTCTTCCAAA |
| β-actin-F | GGGACCTGACTGACTACCTC |
| β-actin-R | TCATACTCCTGCTTGCTGAT |
| **3’UTR cloning Primer** |  |
| HAPLN1 3’UTR-F-XhoI | CCGCTCGAGCGGAGACTTAGAGCCAAAC |
| HAPLN1 3’UTR-R-NotI | ATTTGCGGCCGCTAACTGCCCTGAGTAGATG |
| SFMBT1 3’UTR-F-XhoI | CCGCTCGAGCTGCCAACTGAGAAGGACA |
| SFMBT1 3’UTR-R-NotI | ATTTGCGGCCGCTGGGAGGGAACAGAATGAA |
| ARID4B 3’UTR-F-XhoI | CCGCTCGAGCATAGTTAAGGGGTGGGTC |
| ARID4B 3’UTR-R-NotI | ATTTGCGGCCGCTTGGCAAAGTACAGTAGTTCC |
| BCAT1 3’UTR-F-XhoI | CCGCTCGAGCGTAGAAAGAAATAAACCCTT |
| BCAT1 3’UTR-R-NotI | ATTTGCGGCCGCTGGAATCTGCAAATAATAAAG |
| CTNND2 3’UTR-F-XhoI | CCGCTCGAGCGACAGTGGGCAGCACCTTT |
| CTNND2 3’UTR-R-NotI | ATTTGCGGCCGCTGAAACACGGCGGCATTGGT |
| DCUN1D1 3’UTR-F-XhoI | CCGCTCGAGCAGACTGAGGGTTGAGACA |
| DCUN1D1 3’UTR-R-NotI | ATTTGCGGCCGCTCAAAACACCATTCCAGAT |
| DHX29 3’UTR-F-XhoI | CCGCTCGAGCAATTATCTGAAAATGGGTC |
| DHX29 3’UTR-R-NotI | ATTTGCGGCCGCTTTATTGTAAATGTAGTGGC |
| DOCK9 3’UTR-F-XhoI | CCGCTCGAGCGACGAGCGTCTTACCGAATT |
| DOCK9 3’UTR-R-NotI | ATTTGCGGCCGCTTCCCCTTGGTCCTCCCTGT |
| LGR4 3’UTR-F-XhoI | CCGCTCGAGCGAGGTGGCAGTTTATTTCT |
| LGR4 3’UTR-R-NotI | ATTTGCGGCCGCTCACAGTTCTAGCTGGGACA |
| SOCS7 3’UTR-F-XhoI | CCGCTCGAGCTGCTGGTCACCACCAAGGG |
| SOCS7 3’UTR-R-NotI | ATTTGCGGCCGCTATCCAACGGGCACAAACCC |
| TAC1 3’UTR-F-XhoI | CCGCTCGAGCATGGGCAATGACAGGTAA |
| TAC1 3’UTR-R-NotI | ATTTGCGGCCGCTGAAAACAAATAGGAAAAGCA |
| **3’UTR mutagenesis primer** |  |
| SFMBT1 3’UTR mut-F | GCTGGATCTTTGAATTTTAAATGC |
| SFMBT1 3’UTR mut-R | GATTTGCTGCATTTAAAATTCAAA |
| DCUN1D1 3’UTR mut-F | TCATTCTCGCATAAATTTTAATG |
| DCUN1D1 3’UTR mut-R | ATGAAGACAGCATTAAAATTTAT |
